# *Leonurus cardiaca* L. as a Source of Bioactive Compounds: An Update of the European Medicines Agency Assessment Report (2010)

**DOI:** 10.1155/2019/4303215

**Published:** 2019-04-17

**Authors:** Radu Claudiu Fierascu, Irina Fierascu, Alina Ortan, Ioana Catalina Fierascu, Valentina Anuta, Bruno Stefan Velescu, Silviu Mirel Pituru, Cristina Elena Dinu-Pirvu

**Affiliations:** ^1^University of Agronomic Sciences and Veterinary Medicine of Bucharest, 59 Mărăști Blvd., 011464, Bucharest, Romania; ^2^National Institute for Research & Development in Chemistry and Petrochemistry – ICECHIM Bucharest, 202 Spl. Independentei, 060021, Bucharest, Romania; ^3^University of Medicine and Pharmacy “Carol Davila”, 37 Dionisie Lupu Str., 030167, Bucharest, Romania

## Abstract

*Leonurus cardiaca* L. (motherwort) is a perennial herb, native to Asia and southeastern Europe, with widespread global occurrence in present days. The plant was historically used as cardiotonic and for treating gynaecological afflictions (such as amenorrhea, dysmenorrhea, menopausal anxiety, or postpartum depression). Although its use in oriental and occidental medicine is relatively well documented, the recent progress registered raises the need for an update of the Medicines Agency assessment report on* Leonurus cardiaca* L., herba (2010). The current study presents the progress made within the 2010-2018 timeframe regarding the potential applications and scientific evidences supporting the traditional use of motherwort, in the same time suggesting future research opportunities.

## 1. Introduction

*Leonurus cardiaca* L. (common names –* motherwort* in English,* Echte Herzgespann* – Deutsch,* agripaume* – French, etc) represents a perennial herb belonging to the* Lamiaceae* family. The plants grow up to 1 m, with the hollow aerial stalks growing from the rhizomes. The leaves are palmately lobed, being covered with stiff hairs. Flowers, grouped in 10-20 clusters in the leaf's axils of the last 10-15 knots, are pink and about 1 cm long [1]. The plant, original to Asia and southeastern Europe is now world-spread, due to its medicinal use [2–4]. The potential application in treating several cardiac disorders, as well as female-specific afflictions, made* L. cardiaca* a very good candidate for development of alternative treatments, in both traditional eastern and modern medicine [2, 5]. Besides the traditional medical use, motherwort is used in some cuisines as condiment in various vegetable soup recipes, particularly the lentil or split peas ones, or for flavoring of beer and tea [6], thus increasing the potential intake of the medicinal plant by the general public.

**Figure 1:**
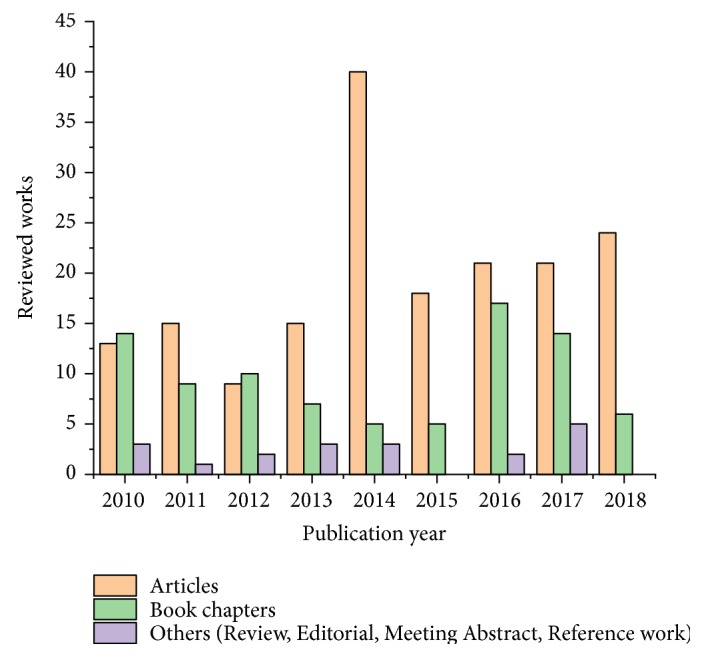
Works published in the time period 2010-2018 including* L. cardiaca*.

The current review intends to present the main findings regarding the composition and main biological activities of* L. cardiaca*, as emerging from the scientific studies published within the 2010-2018 timeframe. The time period was selected in order to complete the very comprehensive “Assessment report on* Leonurus cardiaca* L., herba” published by the European Medicine Agency [5] with the latest findings. The search methodology involved accessing and evaluating the papers found in the PubMed, ScienceDirect, Wiley Online Library, ACS Publications, and SpringerLink databases (search term “*Leonurus cardiaca*”, 27.12.2018). After the removal of duplicate entries, 283 studies were taken into consideration.  Figure 1 describes the distribution of the reviewed works by publication year and type of paper. Most of the information presented in the present work was collected from the “Article” type papers (176 works).

## 2. Composition of* L. cardiaca*

**Figure 2:**
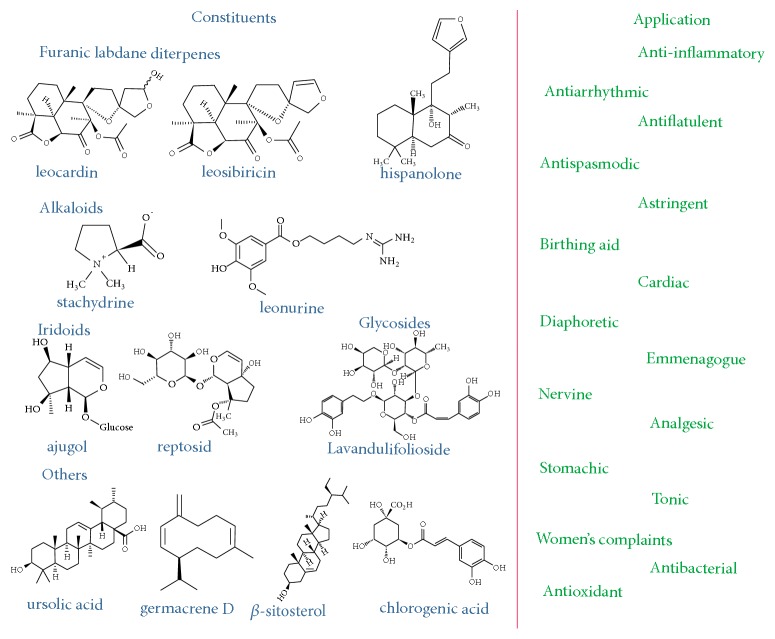
Composition and applications of* L. cardiaca* as emerging from pre-2010 works (adapted from [5]).

The composition of* L. cardiaca* was previously presented by the EMA report [5], consisting of furanic diterpenes (labdanes), alkaloids (of special interest being stachydrine), sterols, iridoids, flavonoids, ursolic acid, minerals, and others.  Figure 2 presents the constituents of* L. cardiaca* and their potential biomedical application, as presented by the pre-2010 literature sources.

The data briefly presented in Figure 2 can be completed with the findings from the time period covered by the present review. Rusch et al. [7] identified in the* L. cardiaca* extract the presence of a chlorinated major iridoid glucoside (7-chloro-6-desoxy-harpagide), confirmed by ESI-MS and 1d/2d 1H/13C NMR. Kuchta et al. [8] quantified by RP-HPLC the presence of ferulic acid, chlorogenic acid, caffeic acid, cichoric acid, rutoside, lavandulifolioside, verbascoside, and isoquercitrin in* L. cardiaca* extract, as well as stachydrine in different parts of* L. cardiaca*, included, for the first time in literature, in the fruits (0.2%) [9, 10].

The leaves essential oil was found to contain caryophyllene, 39.8%; *α*-humulene, 34.8%; *α*-pinene, 5.6%; *β*-pinene, 0.5%; linalool, 0.7%; and limonene, 0.4%, while the ursolic acid present in the leaves was quantified to be 0.26% (dry wt.) [11].

Using HPLC-MS, Zhogova et al. [12] quantified several active compounds in both medicinal plant raw material and medicinal preparation (harpagide, ajugol, galiridoside, harpagide acetate, ajugoside, galiridoside, chlorogenic acid, lavandulifolioside, verbascoside, rutin, hyperoside, isoquercitrin, and apigenin-7-O-glucoside).

An optimized recipe for the extraction of polysaccharides from motherwort leaves was presented by Tahmouzi and Ghodsi [13], obtaining a yield of 9.17 ± 0.39% for 81.4°C extraction temperature, 106.6 min. extraction time, and 45.2 ratio of water to raw material. Although the presence of leonurine in the* L. cardiaca* extracts is presented by some authors, including the previously cited report [5, 14], others support the contrary, not identifying the compound in the* L. cardiaca* extract [15].

## 3. Biological Activities of* L. cardiaca*

The main biological activities of* L. cardiaca* can be divided in several main categories.

### 3.1. Cardiovascular Action

The application of* L. cardiaca* in cardiovascular disorders represents one of the main applications of motherwort products [2]. Ritter et al. [16] evaluated the cardiac and electrophysiological effects of several types of* L. cardiaca* extracts by epicardial potential mapping and by evaluation of the effect on the cardiac ion currents using different types of cell models. The obtained results suggested that* L. cardiaca* extract acts as a mixed I_Ca.L-_ (L-type calcium current), I_Kr-_ (rapid delayed rectifier current) antagonist, and I_f_ (funny current, recorded in sinoatrial node cells from guinea pigs) modulator, supporting its application as an antianginal and antiarrhythmic agent.

The cardioprotective potential of ursolic acid (the natural pentacyclic triterpenoid carboxylic acid commonly found in different* L. cardiaca* formulations) was demonstrated by Liobikas et al. [17]. Their study revealed that the ursolic acid induced uncoupling of oxidative phosphorylation in the heart mitochondria without affecting State 3 respiration rate, in the same time suppressing the H_2_O_2_ production in isolated mitochondria, in a dose-dependent manner. The uncoupling of mitochondrial oxidation from phosphorylation, partial inhibition of the mitochondrial respiratory chain, and a reduction in the generation of free radicals in mitochondria were also observed by Bernatoniene et al. [18] in rat heart mitochondria using* L. cardiaca* ethanol extracts.

The clinical trial conducted by Shikov et al. [19] on fifty patients treated with 1200 mg* L. cardiaca* oil extract per day for 28 days revealed significant changes in systolic blood pressure, diastolic blood pressure, heart rate, and ECG for patients with stages 1 and 2 arterial hypertensions, accompanied by an improvement of psychoemotional status (anxiety, emotional liability, headache, and sleep disorders), especially visible for stage 1 patients.

Stachydrine (alkaloid found in* L. cardiaca*) was proven by Xie et al. [20] to ameliorate homocysteine- (Hcy-) induced endothelial dysfunction via nuclear factor erythroid 2–related factor 2 (Nrf2) dependent upregulation of guanosine triphosphate cyclohydrolase I (GTPCH1) and dihydrofolate reductase (DHFR) enzymes and increase in bioavailabilities of tetrahydrobiopterin (BH4) and nitric oxide (NO), thus protecting endothelial function.

The cardiotonic potential of* L. cardiaca* has been mentioned by Goetz [21, 22], Zaurov et al. [23], Brenyo and Aktas [24], Kidd [25], Jarić et al. [26], Suroowan and Mahomoodally [27], Wang et al. [28], Madridejos Mora [29], Yarnell [30], Orhan et al. [31], Dong et al. [2], and Bianchi [32].

### 3.2. Anti-Inflammatory, Antimicrobial Effect, and Application in Female Disorders

The anti-inflammatory potential of leonurine (considered by some authors a natural component of* L. cardiaca* [5]) on* E. coli*-induced mastitis in mice [33]: their results suggested that leonurine alleviates the histopathological changes, downregulates the levels of proinflammatory cytokines (TNF-*α* and IL-6), upregulates the level of anti-inflammatory cytokine IL-10, and inhibits the expression of nitric oxide synthase (iNOS) and cyclooxygenase-2 (COX-2). The suggested mechanism involves the inhibition of expression of toll-like receptor 4 (TLR4) and nuclear factor-kappa B (NF-*κ*B) activation and mitogen-activated protein kinases (p38), extracellular signal-regulated kinase (ERK), and Jun N-terminal kinase (JNK) phosphorylation. The anti-inflammatory potential of leonurine was also demonstrated by Liu et al. [34] in rat animal models of acute gouty arthritis. The obtained results support the use of leonurine as COX-2, mPGES-1 (microsomal prostaglandin E synthase-1), and 5-LOX (5-lipoxygenase) inhibitor, leading to antiarthritis effects. In the same time, amelioration of monosodium urate crystal-induced inflammation was achieved by decreasing interleukin-1*β* (IL-1*β*) and tumor necrosis factor-alpha (TNF-*α*) production.

The immunomodulatory potential of acetone/water extract of* L. cardiaca* at a 100 *µ*g/ml concentration was assessed by Sadowska et al. [35], revealing a significant reduction of the platelet aggregation in the presence of arachidonic acid, an application that could be beneficial in preventing inflammatory lesions. In the same time, the tested extract did not exhibit proapoptotic activity.

The traditional use of* L. cardiaca* as an anti-inflammatory and antimicrobial agent was also supported by the results presented by Flemmig et al. [36]. They tested several extracts and components of* L. cardiaca* for their ability to regenerate the pseudo-halogenating activity of lactoperoxidase (LPO). The results supported the use of components with a 3,4- dihydroxyphenylic partial structure (such as caffeic acid derivatives or phenylethanoids) as efficient LPO activity regenerators, as well as the use of* L. cardiaca* ethanol extract for the same application. The same study also presents the isolation of the compound caffeoylmalic acid, which also revealed moderate LPO activity-regenerating effects.

Micota et al. [37] studied the antimicrobial potential of* L. cardiaca* acetone-water extract and its component ursolic acid by determining the minimal inhibitory concentration, as well as the antiadhesive and antibiofilm properties against* Staphylococcus aureus* strain (potential etiological agent of infective endocarditis). Their results (MIC = 6 mg/ml for extract and 0.25 mg/ml for ursolic acid) showed weak biostatic activity of* L. cardiaca* extract in comparison to ursolic acid, but both preparations possessed antiadhesive potential. The* S. aureus* biofilm formation was slightly inhibited by the extract (5%), but strongly inhibited by ursolic acid (85%) at concentrations of 3/4 MIC.

The study of Samoilova et al. [38] on the effect of subinhibitory doses of plant extract (including* L. cardiaca*) on* Escherichia coli* biofilm formation revealed that motherwort extract showed a synergistic effect with sublethal concentration of streptomycin (30 *µ*g/ml), inhibiting the specific biofilm formation. As the study was focused on the capacity of low concentration plant extracts and polyphenols to induce adaptive mechanisms in* E. coli*, it cannot be considered a truly antimicrobial study.

Micota et al. [39] used subinhibitory doses of* L. cardiaca* extract to establish its effects on the characteristics of* Staphylococcus aureus*. The beneficial effect of the extract was observed, such as reduction in staphylococcal adherence, aggregates formation, coagulase activity, protein A expression, or alpha-toxin synthesis. However, some of their findings (e.g., enhancement of staphylococcal tolerance to exogenous hydrogen peroxide after preincubation with the extract) led to the conclusion of a possible risk of adverse effects.

Wu et al. [40] presented the application of leonurine for ameliorating the inflammatory responses in endometritis model in mice. The leonurine treatment suppressed the TNF-*α* and IL-1*β* mRNA levels in uterus tissues, inhibited lipopolysaccharide-induced TLR4 expression, and reduced the phosphorylated p65 and I*κ*B*α* proteins.

The clinical trial conducted by Denham et al. [41] documented the herbal prescribing in usual practice, covering a total of 80 herbs on 141 prescriptions (the most encountered being* L. cardiaca* - 77%) for treatment of symptoms associated with the menopause on 35 subjects.* L. cardiaca* was mainly prescribed to control hot flushes, as a gynecological tonic and as a relaxant.

In a randomized study conducted on 165 women undergoing cesarean section, motherwort, in combination with oxytocin, proved to be efficient for preventing postpartum hemorrhage [42].

Regarding the use of* L. cardiaca* in female disorders, the plant is listed as a natural remedy for female reproductive system (anxiolytic, antispasmodic, PMS, and menopausal anxiety) [43], as an emmenagogue, nervine, amenorrhea, analgesic, and uterine astringents/vascular decongestants and for treating adolescent dysmenorrhea [44], for treating menopausal anxiety, and as tranquilizer [45]. Lans et al. [46] present motherwort as a natural cure used in North America from colonial times, due to its tonic, emmenagogue, antispasmodic, and nervine properties, citing pre-1900 works.

### 3.3. Antioxidant Action

The antioxidant activity of* L. cardiaca* products was evaluated using several methods. Sadowska et al. [35] evaluated the antioxidant potential of* L. cardiaca* extract by ABTS^*∙*^, DPPH^*∙*^, and ferric reducing antioxidant power assay, obtaining values of the antioxidant capacity in the range 350±20–455±17 *µ*M Trolox/g. Ebrahimzadeh et al. [47] evaluated the antioxidant potential of Iranian native* L. cardiaca* extract obtained from dried aerial parts by percolation using methanol, by comparison with* Grammosciadium platycarpum* and* Onosma demawendicum* extracts. The results obtained by DPPH^*∙*^ assay (IC_50_ = 144 ± 12.1 mg/ml), iron reducing assay (results superior to vitamin C in the concentration range 25-100 *µ*g/ml), nitric oxide-scavenging assay (IC_50_ = 0.15 ± 0.01 mg/ml), metal chelating assay (IC_50_ = 20 ± 1 *µ*g/ml), and scavenging of hydrogen peroxide (IC_50_ = 438.2 ± 21.8 *µ*g/ml) were correlated with the total phenol content (54.3 ± 1.8 mg gallic acid equivalent/g of extract). Ebrahimzadeh et al. [48] evaluated the correlation between the total phenolic compounds and total flavonoids content and the nitric oxide scavenging properties for 26 Iranian medicinal plants. The authors found good correlation between total phenolic content (54.3 ± 2.71 mg gallic acid equivalent/g of extract) for* L. cardiaca* aerial parts methanol extract, total flavonoids content (35.2 ± 1.76 mg quercetin equivalents/g of extract), and nitric oxide radical scavenging activity (IC_50_ = 0.15 ± 0.007 mg/ml).

Armatu et al. [49] evaluated the antioxidant potential of several extracts obtained from Romanian Lamiaceae species (including* L. cardiaca* methanol extract) using the DPPH^*∙*^ assay, phosphomolybdenum method, and chemiluminescence assay in relationship with HPTLC fingerprints and total phenolic content. The results obtained by the three antioxidant assays at 5 mg/ml concentration (DPPH^*∙*^ – 20%, total antioxidant capacity –approx. 40 mg ascorbic acid equivalents/g and 48% antioxidant activity for the chemiluminescence activity) were correlated with the relatively low total phenolics content (2.8 mg gallic acid equivalents/g of extract).

Jafari et al. [50] evaluated the total phenolic content and antioxidant capacity (DPPH^*∙*^ assay) of different fractions of Iranian* L. cardiaca* extract. The best results were obtained for the 50:50 metanolic-aqueous fraction (total phenolic content 70.79±4.41 gallic acid equivalents/g of fraction and IC_50_ = 53.79 *µ*g/ml – DPPH^*∙*^ assay).

The influence of drying method was studied by Yi and Wetzstein [51] using three drying methods (greenhouse sun-drying, 40°C oven-drying, and 70°C oven-drying) on 80% methanol and 80% ethanol extracts from the leaves of cultivated* L. cardiaca* plants. The best results were obtained for ethanol extracts of 40°C oven-dried plants (total polyphenolics – approx. 70 mg/g GAE, Trolox-equivalent antioxidant capacity – approx. 400 mM/g TE).

Polysaccharides from* L. cardiaca* extract exhibited a very good scavenging activity of hydroxyl radicals (IC_50_ = 6.98 ± 0.87 mg/mL), compared with vitamin C (IC_50_ = 7.59 ± 0.94 mg/mL) [13]. Wong et al. [52] evaluated the antioxidant potential and phytochemical composition of aqueous extracts obtained from Malaysian plants. The extract showed a relatively good antioxidant potential (>60% DPPH^*∙*^ scavenging activity at 16 mg/ml, >80% NO scavenging activity at 10 mg/ml, and >85% metal chelating activity at 10 mg/ml) for a phytochemical composition of 12.13 ± 0.12 mg GAE/g dry weight (total phenolics), 9.86 ± 0.15 mg QE/g dw (total flavonoids), and 2.01 ± 0.01 mg CAE/g dw (hydroxycinnamic acids). The study of Ji et al. [53] on the antioxidant effect of plants with therapeutic potential on gynecological diseases revealed no significant influence on the lag phase duration of copper-induced low-density lipoprotein cholesterol (LDL-C) oxidation, the authors suggesting as main reason for the lack of antioxidant activity the solvent used for extraction. Ziyatdinova et al. [54] proposed an alternative method for the evaluation of the antioxidant activity (determined as DPPH^*∙*^ inhibition using differential pulse voltammetry) and compared the values obtained for several medicinal plants with those obtained spectrophotometrically. The results showed better antioxidant effect for the* L. cardiaca* infusion (32±1%, determined by differential pulse voltammetry, respectively, 33±1%, determined by spectrophotometry) compared with the tincture (70% ethanol, 16.6±0.4%, determined by differential pulse voltammetry, respectively, 17±2% determined by spectrophotometry). Ziyatdinova et al. [55] described a chronocoulometric method for the evaluation of the antioxidant potential of 42 commercial-available medicinal plants tinctures (including* L. cardiaca*), also establishing a correlation between the antioxidant potential and the total phenolics content. The evaluation revealed a relatively low antioxidant capacity for the* L. cardiaca* tincture (1.7±0.02 mg quercetin/mL).

The antioxidant potential of* L. cardiaca* extracts was also briefly presented in other works (for example, the works of Sen and Chakraborty [56] and Krishnaiah et al. [57]).

### 3.4. Other Applications

Motherwort was historically used for the treatment of several nervous afflictions, such as depression, anxiety, or stress [57]. Romm [58] classified* L. cardiaca* as a natural remedy for the treatment of several afflictions, including postpartum depression, while the traditional internal use of motherwort for the treatment of epilepsy was documented by Adams et al. [59]. The potential towards the treatment of anxiety and depressive disorders was evaluated by Rauwald et al. [15, 60], by studying the effect of* L. cardiaca* extract and constituents (isoleosibirin, 7R-chloro-6-desoxy-harpagide, lavandulifolioside, stachydrine, and leonurine) on the neuronal receptor gamma-aminobutyric acid (GABA). The extract inhibited the concentration-dependent binding of [(3)H]-SR95* *531 to the GABA site of the GABA type A receptor with a binding affinity (IC_50_) of 21 *µ*g/ml, suggesting a potential neurological mechanism of action of* L. cardiaca*, based on interaction to the GABA site of the GABA type A receptor. The individual components tested (except leonurine – IC_50_ - 15 *µ*g/ml) did not exhibit significant activity. Commercially available leonurine was demonstrated by Xu et al. [61] to ameliorate LPS-induced acute kidney injury in mice. The nephroprotective effect was expressed, after 14 days of treatment, by the values of reactive oxygen species (ROS), malonyldialdehyde (MDA), and reduced glutathione (GSH) which were reduced to near control levels, while the lipopolysaccharide-induced tubular damage was significantly ameliorated, decreased renal injury biomarker (KMI-1), and inhibited the nuclear transfer of NF-*κ*B p65. A similar nephroprotective effect was registered by Cheng et al. [62] in mouse unilateral urethral obstruction, by suppressing ROS-mediated TGF-*β*/Smad3-induced tubulointerstitial fibrosis and inhibiting NF-*κ*B-mediated inflammatory response. Leonurine was also found to ameliorate cognitive disfunction in rats' model [63]. At a 100 concentration, it was found to decrease the oxygen-glucose deprivation- (OGD-) induced brain cell death to approx. 130% (fold of control group), an approx. 50% reduction, compared with the OGD group. Also, leonurine was found to alleviate the impaired spatial learning and memory, as demonstrated through Morris water maze test. Leonurine also decreased the concentrations of glutamate and hydrogen peroxide in hippocampus, ameliorated the impaired long-term depression in hippocampus, improved cognitive function by modulating the N-methyl-D-aspartate receptors-associated proteins, and protected rats from bilateral carotid artery occlusion-induced damage by inhibiting autophagy. The results suggested leonurine as a potential drug candidate for chronic cerebral hypoperfusion.

Ethanolic extract obtained from aerial parts of* L. cardiaca* was evaluated by Rezaee-Asl et al. [64] as a potential analgesic, using formalin, tail flick, and hot plate tests in mice. The results of the study proved that at 500 mg/kg the extract was able to reduce the formalin-induced pain in the early phase, increase the antinociceptive activity, and significantly influence the reaction time of the animals to the hot plates, supporting the analgesic properties of the extract, action mediated through peripheral, and central inhibitory mechanisms.

The antiviral potential of* L. cardiaca* was reviewed by Todorov et al. [65]. Different components of the extract were found to be active against several types of viruses (ursolic acid - HCV, HPV-18; quearcetin - HSV-1, poliovirus 1, RSV; hyperoside – DHBV; apigenin – HSV-2; rutin – HIV-1), while the chloroform and methanol extracts were presented to possess antiherpes activity against HSV-1 and 2.

Onumah [66] presents* L. cardiaca* as a potential adjuvant in treating overactive thyroid, due to its action against symptoms associated with hyperthyroidism (palpitations and anxiety). Inhibition of the thyroid-stimulating hormone and the reduction of excess production of thyroid hormones were also presented by Shokri et al. [67], the property being assigned to its content in rosmarinic acid.

Unlike many other plant species, the evaluated literature data (even outside the time period covered by the present review) presents no research regarding the phytosynthesis of metallic nanoparticles using* L. cardiaca* extracts, although the procedure was presented for* L. japonicus* [68].

Table 1 summarizes the main biological activities presented.

## 4. Dosage and Toxicology

As previously has been presented,* L. cardiaca* preparations are currently used in the treatment of several conditions. Relative to commercial products, the EMA report cited [5] presents them to be safe, suggesting a duration of use limited to four weeks. The report also presents the adverse effects of an intake of 3.0 grams of a powdered extract per day (diarrhea, uterine bleeding, and stomach irritation).* L. cardiaca* is listed as a “herb to avoid during pregnancy” [69, 70], mainly due to its emmenagogue and uterine stimulation properties. Kaye et al. [71] listed* L. cardiaca* as a “herbal drug associated with bleeding abnormalities”, an aspect to be considered by the anesthesia practitioner.

Anadón et al. [72] presented the reduction of platelet aggregation and fibrinogen levels upon intravenous administration of motherwort.* L. cardiaca* also potentiates antithrombotic and antiplatelet effects, increasing the risk of bleeding. When administered concomitant with benzodiazepines, motherwort can also have a synergistic sedative effect resulting in coma [73].

*L. cardiaca* was also presented to potentialize the effect of warfarin, by inhibiting platelet aggregation [74].

Related to individual compounds, data are scarce and mainly outside the current review time period: Milkowska-Leyck et al. presented moderate toxicity of lavandulifolioside (LD_50_ approx. 1000 mg/kg) and* n-*butanol extract (LD_50_ approx. 400 mg/kg) for intravenous administration, while for oral administration, the toxicity was much lower (LD_50_ >2000 mg/kg) [75]; Mitchell and Rook [76] present the potential of* L. cardiaca* leaves to cause photosensitization and dermatitis; herbal intravenous injection has a LD_50_ of 30-60 mg/kg (mice), while the intravenous LD_50_ of the total alkaloids of the herb was approx. 570 mg/kg, while the minimal lethal dose of leonurine in frogs (subcutaneous administration) was 400-600 mg/kg [77]. Due to the lack in scientific evidences, most of the sources presenting traditional use of motherwort suggest the strict following of relevant directions on products containing* L. cardiaca* and the requesting supplemental information from pharmacists, physician, or other healthcare professionals before use [78]. Some chemical components from* L. cardiaca* aerial parts (pyrrolidine alkaloids, such as stachydrine, cyclic peptide, such as cycloleonurinine or labdane diterpenes, such as leosibiricin) are considered “chemicals of concern” for human health when used in food and food supplements [79].

## 5. Conclusions

The use of motherwort (*Leonurus cardiaca* L.) has been documented since ancient times, especially as a cardiotonic and for the treatment of gynecological conditions. The composition (dominated by furanic diterpenes, alkaloids, sterols, and iridoids) was proved to present a complex biological activity, with cardioprotective, antioxidant, antimicrobial, anti-inflammatory, analgesic, nephroprotective, and antiviral properties, among others.

The current study aimed to present the progress made in the study of motherwort from the date of the European Medicine Agency “*Assessment report on Leonurus cardiaca L., herba*”. According to our findings, most of the literature data focuses on cardioprotective and antioxidant potential of* L. cardiaca*, although the data also suggest the exploration of new applications. This, in turn, would be a promising research area for future studies. Future research should also be focused on a definitive conclusion regarding the composition of motherwort (especially the presence of leonurine), as well as the opening of new research directions, such as the use of* L. cardiaca* extracts in nanotechnology (for the phytosynthesis of nanoparticles).

## Figures and Tables

**Table 1:** Biological activities of *L. cardiaca* (2010-2018).

Origin	Part of plant/product	Type of paper	Activity	Tests performed	Components responsible for activity	Ref.
-	Aerial parts	Review	Against uterine infection or other gynecological diseases, tachyarrhythmia and other cardiac disorders	-	-	[2]
-	Aerial parts	Review	Antioxidant	-	Rutin and derivatives of hydroxycinnamic acid	[2]
Commercially available, Germany	Aerial parts extract (aqueous Soxhlet) and refined extracts: dichloromethane, 70% hydroethanolic	Research	Antianginal and antiarrhythmic	Epicardial potential mapping, effect on the cardiac ion currents	phenolic constituents	[16]
Commercially available	Ursolic acid	Research	Cardioprotective	Evaluation of mitochondrial respiratory rates, mitochondrial H_2_O_2_ generation, H_2_O_2_ antioxidant activity	-	[17]
Wild-growing, Lithuania	70% ethanol extract	Research	Cardioprotective	Measurement of the mitochondrial respiration rate and mitochondrial H_2_O_2_ generation	Chlorogenic acid and flavonoids orientin, quercetin, hyperoside, and rutin	[18]
-	Soybean oil extract	Clinical trial	Treatment of arterial hypertension accompanied by anxiety and sleep disorders	dynamics of psychoneurological symptoms; state – activity – mood, the Clinical Global Impression scale, systolic blood pressure, diastolic blood pressure, heart rate and ECG	Iridoids	[19]
Commercially available	Stachydrine	Research	endothelial function protection	Determination of cell viability, Nitric oxide assay, Measurement of BH4, Measurement of cGMP levels in rat arterial rings. Measurement of vasorelaxation, Quantitative reverse transcriptase-PCR (qRT-PCR), Western blotting	-	[20]
-	Extract	Review	Sedative, cardiotonic	-		[21]
-	Aerial parts extract	Review	cardiotonic bradycardic agent, hypotensive	-	-	[22]
-	Aerial parts tincture	Review	Sedative, decreases arterial pressure and strengthens the contraction of uterus muscles	-	-	[23]
-	Stachydrine	Review	Protective effect in experimental myocardial ischemia reperfusion injury	-	-	[23]
-	-	Review	Sedative, antispasmodic, electrophysiologic			[24]
-	Lavandulifolioside	Review	chronotropic effects	-	-	[24]
-	-	Review	Cardiovascular	-	-	[25]
-	Aerial parts, tea	Review	Strengthening the heart, arrhythmia, antihypertensive	-	-	[26]
-	-	Review	Cardiac arrhythmias, tachycardia, heart palpitations	-	phenylpropanoid glycosides	[27]
-	Aerial parts	Review	Cardioprotective, antioxidant	-	-	[28]
-	-	Review	relief of nervous tension symptoms, treatment of arrhythmias	-	-	[29]
-	-	Review	maintenance of normal cardiac rhythm	-	-	[30]
-	-	Review	Cardiotonic	-	-	[31]
-	-	Review	Sedative, vasodilator	-	-	[32]
Commercially available	Leonurine	Research	Anti-Inflammatory	Histopathological analysis, Cytokines Analysis, Quantitative Real-Time Polymerase Chain Reaction, Western Blot Analysis	-	[33]
Commercially available	Leonurine	Research	Agent for gouty arthritis treatment	Histological examination, Lentiviral transduction, Western blot analysis, Cytokine measurement	-	[34]
Commercially available, Poland	Acetone-water (70:30, v/v) extract	Research	Immunomodulatory, antioxidant	NO production in Human umbilical vein endothelial cells, platelet aggregation, ABTS^*∙*^, DPPH^*∙*^ and FRAP assay	-	[35]
Commercially available, Germany	Ethanol extract (70%), Soxhlet extraction, Methanol extract, other phases	Research	Anti-inflammatory and antimicrobial	Effect of extracts and single components on lactoperoxidase activity	Phenolic components with 3,4-dihydroxyphenyl partial structure	[36]
Commercially available, Poland	Leaves acetone-water (70:30, v/v) extract	Research	Antimicrobial	Evaluation of antimicrobial, anti-adhesive and anti-biofilm properties against *S. aureus*	Iridoid glycosides, di- and triterpenoids, flavonoids, tannins and volatile oils	[37]
Commercially available, Russia	Aerial parts water extract	Research	Anti-biofilm formation	Biofilm formation assay	-	[38]
Commercially available, Poland	Aerial parts polyphenol-enriched extract	Research	Effect of sub-inhibitory concentration extracts on *S. aureus*	*S. aureus *survival, staphylococcal tolerance to oxidative stress, *S. aureusα*-toxin (Hla) release and protein A (SpA) expression, Staphylococcal aggregation in human plasma, Fibrinogen polymerization and *S. aureus*adhesion to fibrin	-	[39]
Commercially available	Leonurine	Research	Anti-inflammatory	Histopathological analysis, Cell viability assay, Analysis of cytokines, qRT-PCR analysis, immunoblotting analysis,	-	[40]
-	Aerial parts, tea	Clinical trial	To control hot flushes, gynaecological tonic, relaxant	-	-	[41]
-	Motherwort injection	Trial	Preventing postpartum hemorrhage	Mean blood loss, postpartum hemorrhage, mean systolic blood pressure, diastolic BP, heart rate, respiratory rate, hemoglobin and platelet count, incidence of postpartum hemorrhage, safety assessment	-	[42]
-	Aerial parts	Review	Anxiolytic, antispasmodic, PMS, and menopausal anxiety	-	-	[43]
	Aerial parts, tea, tincture, infusion	Review	Antispasmodic, anti-inflammatory anxiolytic, uterine tonic,	-	-	[44]
-	Aerial parts, tincture, tea, infusion	Review	Menopausal anxiety, insomnia, palpitations hyperthyroidism,	-	-	[45]
-	Aerial parts, infusion, decoction	Review	Tonic, Amenorrhoea, suppressed lochia, dysmenorrhoea, antispasmodic, nervine, emmenagogue	-	-	[46]
Wild-growing, Iran	Aerial parts, methanol extract	Research	Antioxidant, radical scavenging	DPPH radical-scavenging activity, reducing power, nitric oxide-scavenging activity, Metal chelating activity, ferric thiocyanate assay, Scavenging of hydrogen peroxide	Phenolic compounds	[47]
Wild-growing, Iran	Aerial parts, methanol and water extracts	Research	Antioxidant	Nitric oxide radical scavenging activity	Phenolic compounds, flavonoids	[48]
Cultivated, Romania	Aerial parts, methanol extract	Research	Antioxidant, free scavenging potential	phosphomolybdenum reduction assay, DPPH^*∙*^ assay, Chemiluminescence assay	Polyphenolics	[49]
Cultivated, Iran	Aerial parts, extracts and fractions	Research	Antioxidant	DPPH^*∙*^ assay	Phenolic compounds	[50]
Cultivated, Greece	Leaves, 80% methanol or 80% ethanol	Research	Antioxidant	ABTS^*∙*^ assay	Phenolic compounds	[51]
Wild-growing, Iran	Leaves, polysaccharides extract	Research	Antioxidant, antimicrobial	Hydroxyl radical scavenging capacity, DPPH^*∙*^ assay, antimicrobial effect evaluated by the filter disk diffusion plate method against bacteria, yeast and fungi	Polysaccharides	[13]
Wild-growing, Malaysia	Aerial parts, water extract	Research	Antioxidant, antidiabetic	DPPH^*∙*^ assay, nitric oxide radical scavenging assay, metal chelating activity, Alpha-glucosidase inhibition assay	Phenolic compounds	[52]
Commercially available, China	Aerial parts, aqueous extract	Research	Antioxidant	LDLc oxidation delay	-	[53]
Commercially available, Russia	Commercial tinctures and infusions	Research	Antioxidant	DPPH^*∙*^ assay, voltammetry and spectrophotometric	-	[54]
Commercially available, Russia	Commercial tinctures	Research	Antioxidant	Chronocoulometry	Lavandulifolioside, phenolic acids, caffeic acid 4-rutinoside, tannins	[55]
-	-	Review	Antioxidant	-	-	[56]
-	Methanol extract	Review	Antioxidant	DPPH^*∙*^ assay	Flavonoid and phenolic glycosides	[57]
-	Aerial part, tincture, tea	Review	Nervine relaxant, anxiolytic,	-	-	[58]
-	-	Review	Epilepsy treatment	-	-	[59]
Commercially available, Germany	Aerial parts, 45% ethanol extract, Stachydrine, Leonurine, Lavandulifolioside, Isoleosibirin, 7R-chloro-6-desoxy-harpagide	Research	Treatment of anxiety, depression, nervousness, sedative	*In vitro* GABA receptor binding assays,	-	[15, 60]
Commercially available	Leonurine	Research	Nephroprotective	Measurement of TNF-*α*, IL-1, IL-6, IL-8, Measurement of serum creatinine and blood urea nitrogen, Assay of GSH, Assay of ROS, Assay of MDA level, Western blot analyses, Protein assay, Histological assay	-	[61]
Commercially available	Leonurine	Research	Nephroprotective	Measurement of TGF-*β*, TNF-*α*, IL-6 and IL-1*β* level, ROS assay, Assay of MDA level, Assay of GSH level, Western blot analyses, Protein assay, Histological assay	-	[62]
Commercially available	Leonurine	Research	Neuroprotective	Evaluation of spatial learning and memory performances of rats, levels of glutamate and H_2_O_2_ of hippocampus, Western blot assay	-	[63]
-	Aerial parts, ethanolic extract	Research	Analgesic	Nociceptive Behavioral Tests	-	[64]
-	Aerial parts, different types of extracts	Review	Antiviral	-	ursolic acid, quercetin, hyperoside, apigenin-7- glucoside, rutin	[65]
-	-	Review	Treatment of hyperthyroidism, palpitations, anxiety	-	-	[66]
-	-	Review	Treatment of hyperthyroidism	-	Rosmarinic acid	[67]
